# Combination of the G-8 Screening Tool and Hand-Grip Strength to Predict Long-Term Overall Survival in Non-Small Cell Lung Cancer Patients Undergoing Stereotactic Body Radiotherapy

**DOI:** 10.3390/cancers13133363

**Published:** 2021-07-05

**Authors:** Kristian Kirkelund Bentsen, Olfred Hansen, Jesper Ryg, Ann-Kristine Weber Giger, Stefan Starup Jeppesen

**Affiliations:** 1 Department of Oncology, Odense University Hospital, 5000 Odense, Denmark; olfred.hansen@rsyd.dk (O.H.); stefan.jeppesen@rsyd.dk (S.S.J.); 2 Department of Clinical Research, University of Southern Denmark, 5000 Odense, Denmark; jesper.ryg@rsyd.dk (J.R.); ann-kristine.weber.giger@rsyd.dk (A.-K.W.G.); 3 Academy of Geriatric Cancer Research (AgeCare), Odense University Hospital, 5000 Odense, Denmark; 4 Department of Geriatric Medicine, Odense University Hospital, 5000 Odense, Denmark

**Keywords:** non-small cell lung cancer (NSCLC), stereotactic body radiotherapy (SBRT), Geriatric 8 (G-8), hand-grip strength test (HGST), long-term overall survival (OS)

## Abstract

**Simple Summary:**

As the world’s population ages, the number of older patients diagnosed with non-small cell lung cancer will increase, and more patients are expected to suffer from comorbidities. Stereotactic body radiation therapy presents a curative modality and is the treatment of choice for patients with localized non-small cell lung cancer that is considered medically inoperable (often due to multimorbidity). However, in these patients, age-related comorbidities—rather than lung cancer—are the leading cause of mortality. To address age-related comorbidities and optimize the general health status of older patients with cancer, the Geriatric 8 screening tool was developed. However, the Geriatric 8 is mainly based on nutritional assessment; therefore, we aimed to evaluate if a combination of the Geriatric 8 and a test of physical functioning, such as hand-grip strength, can lead to more accurate identification of patients who are in need of geriatric care than the Geriatric 8 alone.

**Abstract:**

The Geriatric 8 (G-8) is a known predictor of overall survival (OS) in older cancer patients, but is mainly based on nutritional aspects. This study aimed to assess if the G-8 combined with a hand-grip strength test (HGST) in patients with NSCLC treated with stereotactic body radiotherapy can predict long-term OS better than the G-8 alone. A total of 46 SBRT-treated patients with NSCLC of stage T1-T2N0M0 were included. Patients were divided into three groups: fit (normal G-8 and HGST), vulnerable (abnormal G-8 or HGST), or frail (abnormal G-8 and HGST). Statistically significant differences were found in 4-year OS between the fit, vulnerable, and frail groups (70% vs. 46% vs. 25%, *p* = 0.04), as well as between the normal and abnormal G-8 groups (69% vs. 39%, *p* = 0.02). In a multivariable analysis of OS, being vulnerable with a hazard ratio (HR) of 2.03 or frail with an HR of 3.80 indicated poorer OS, but this did not reach statistical significance. This study suggests that there might be a benefit of adding a physical test to the G-8 for more precisely predicting overall survival in SBRT-treated patients with localized NSCLC. However, this should be confirmed in a larger study population.

## 1. Introduction

As the world’s population ages, the number of people diagnosed with cancer will increase, and more patients are expected to suffer from comorbidities that will impact mortality [1,2]. Almost 50% of all patients diagnosed with localized non-small cell lung cancer (NSCLC) are older than 70 years of age [3], one-fourth of whom are considered medically inoperable due to age-related comorbidities, functional status, or refusal of surgery [4]. For these patients, stereotactic body radiation therapy (SBRT) is a curative treatment modality that is considered the treatment of choice [5,6].

Aging is a heterogeneous process, and patients of the same chronological age may reflect different physiological ages [7]. A recent study of the survival of SBRT-treated patients with localized NSCLC indicated age-related comorbidities (heart disease, chronic obstructive pulmonary disease, or thromboembolic events)—rather than lung cancer—as the leading cause of mortality [8], emphasizing the importance of addressing comorbidities when planning or evaluating the treatment of oncological patients. Throughout time, screening tools have been developed to predict overall survival (OS) and identify frailty in older patients with cancer. The Geriatric 8 (G-8) screening tool is one of the most extensively studied screening tools and has been demonstrated to be a strong independent predictor of OS [9,10,11]. It is recommended by the International Society of Geriatric Oncology to perform a G-8 screening in older patients with cancer to assess the need for a comprehensive geriatric assessment (CGA) [12]. A G-8 cut-off value of 14, as originally suggested by Bellara et al., has traditionally been used, but an optimal cut-off value when assessing OS in older patients with cancer still has to be decided upon [13,14,15]. The G-8 is derived from the Mini Nutritional Assessment Short-Form questionnaire and focuses mainly on nutritional aspects [16]. However, in older patients with cancer, not only nutritional status but also physical functioning is an important domain to be included when predicting patient-related outcomes [17]. 

The hand-grip strength test (HGST) is a well-established indicator of muscle strength among older patients and is considered the simplest and least complicated instrumented muscle strength measurement [18]. The HGST has been identified as a robust predictor of mortality and to have a statistically significant impact on OS in a G-8-abnormal population of older patients with cancer [11]. A combination of the G-8 and HGST might engulf the essential aspects of both the nutritional and physical domains and give a better prediction of OS in older patients with cancer than that of the G-8 alone. This study aimed to assess if the G-8 combined with HGST in patients with NSCLC and treated with SBRT can predict long-term OS better than the G-8 alone.

## 2. Materials and Methods

This study presents prospectively collected long-term 4-year overall survival data from a single institutional randomized study performed in 2015–2016. The primary aim of the randomized study was to investigate the impact of CGA and interventions as needed on quality of life (QoL), and it was reported in a peer-reviewed and published paper [19]. The standard arm of the randomized study was SBRT without CGA, and the intervention arm was SBRT with CGA. Both treatment arms received the standard best practice of care. 

This study focuses solely on the exploratory aim of investigating the role of the G-8 and HGST for predicting overall survival in patients receiving SBRT for localized NSCLC.

### 2.1. Study Population

Between January 2015 and June 2016, 51 patients with histologically/cytologically proven T1-2N0M0 NSCLC who were referred to the Department of Oncology, Odense University Hospital for treatment with SBRT were included in the randomized study. The patients had either refused or were considered too frail for surgery based on a multidisciplinary assessment. Patients with concomitant malignancies or histories of other malignancies within the last five years were not eligible for the study. Four patients discontinued their participation in the study at randomization because they considered the CGA overwhelming. In total, 47 patients were included. 

All data for the exploratory aim focusing on the G-8 and HGST were analyzed in October 2020, when 29 patients had died.

### 2.2. Study Approach

In the randomized study, all patients received volumetric modulated arc therapy (VMAT) with SBRT 45-66 Gy/3 fractions (F) within nine days and were assigned in a ratio of 1:1 to receive CGA or not. At baseline, an oncologist assessed the Eastern Cooperative Oncology Group performance status (ECOG-PS), Charlson Comorbidity Index (CCI), and the G-8 screening tool, and a nurse assessed the Barthel-20, HGST, and chair-stand test. Toxicity was evaluated at baseline and follow-up by an oncologist using the CTCAE 4.0.

For the exploratory aim of the study, the patients were divided into three groups: fit (normal G-8 and HGST scores), vulnerable (abnormal G-8 or HGST scores), and frail (abnormal G-8 and HGST scores). As a reference for further analysis, patients were also divided into two (normal >14 or abnormal ≤14) and three (high >14, intermediate 11–14, or low <11) groups based solely on their G-8 scores.

#### 2.2.1. Geriatric 8

The G-8 screening tool was developed to identify geriatric risk profiles in older patients with cancer [13]. It consists of eight items: seven items from the Mini Nutritional Assessment (MNA) questionnaire and an additional categorization of self-perception of health [13]. The items from the MNA included questions regarding nutritional status, weight loss, body mass index, motor skills, psychological status, and the number of medications. G-8 scores ≤14 were considered abnormal [13].

#### 2.2.2. Hand-Grip Strength Test

The HGST was measured in kg by using a Smedley dynamometer. Each patient was shown the correct handling and positioning of the instrument according to the clinical assessment recommendations of the American Society of Hand Therapists [20]: elbow in a 90° position with the upper arm tight against the trunk. Measurements were repeated in a series of three on each hand, alternating with short pauses. The mean and maximum values achieved were registered for both hands. Maximum scores for the HGST of <21 kilograms (kg) for men and <15 kg for women were considered abnormal [21]. 

#### 2.2.3. Chair-Stand Test

In the chair-stand test, the patients were asked to sit in the middle of the seat with their back straight, their feet shoulder-width apart and flat on the ground, and their arms crossed at the wrists and held against the chest. In 30 s, the number of full stands was registered. A score of <10, which indicated a high risk of falling, was considered abnormal [22].

#### 2.2.4. Eastern Cooperative Oncology Group Performance Status, Charlson Comorbidity Index, and Barthel-20

The ECOG-PS is used by oncologists to quantify well-being and activities of daily living in order to guide oncological treatment decisions. Patients with ECOG-PS scores of ≥2 were considered frail [23]. The CCI is used for categorizing comorbidities with scores of 0–1, 2–3, and >3, which are considered normal, medium, and high, respectively [24]. The Barthel-20 is used by geriatricians to measure performance in activities of daily living, with scores of ≤18 indicating increased disability [25].

### 2.3. Statistical Methods

No sample size and power calculations were performed due to the exploratory nature of the study. The baseline characteristics of the three groups (fit, vulnerable, and frail) were compared by using univariate analyses. The means/medians were compared by using an unpaired *t*-test or a one-way analysis of variance (ANOVA). Group proportions were compared by using Fisher’s exact test or a Chi-square test.

The survival rates were calculated from the first day of SBRT, with the OS being defined as the time to death from any cause, including lung cancer. The CSS was defined as the time to death caused by NSCLC. The survival analyses were performed by using the Kaplan–Meier method, with testing of the differences using the log-rank test. 

A multivariable Cox regression model was constructed by using the forward method. Since sex, age, histology, and CGA are known predictors of OS, they were forced into the regression analysis. Furthermore, variables with a univariate analysis *p*-value of <0.25 were considered potential predictors of OS and were thus included in the final model. Interaction analyses between variables were performed. In all analyses, a two-tailed *p*-value of <0.05 was considered statistically significant.

## 3. Results

Forty-seven patients were included, and baseline HGST data were missing in one patient. The total study population of 46 patients included ten patients who were considered fit, 28 patients who were considered vulnerable, and eight patients who were considered frail. Of all patients, 76% had abnormal G-8 scores and 24% had abnormal HGST scores.

The median potential follow-up time for the total study population was 60 months (50–68 months). The baseline characteristics of the study population are presented in Table 1. Forty-two patients were considered medically inoperable (23 due to poor pulmonary capacity and 19 due to advanced age, poor performance status, excessive alcohol use, or comorbidity), and four patients declined surgery. Twenty-four patients developed grade 1 toxicity, 39 patients developed grade 2 toxicity, six patients developed grade 3 toxicity, and no patients experienced grade 4 or 5 toxicity. The most common types of grade 3 toxicity were pain for three patients, dyspnea for two patients, and fatigue for one patient.

A statistically significant difference was found between the ECOG-PS scores of patients with normal vs. abnormal G-8 scores. No statistically significant differences in the number of patients who received CGA or other baseline characteristics were found between the G-8 groups or between the fit, vulnerable, or frail combination groups. Other baseline patient characteristics of the G-8 groups and the G-8 and HGST combination groups are presented in Table A1 in Appendix A.

**Figure 1:**
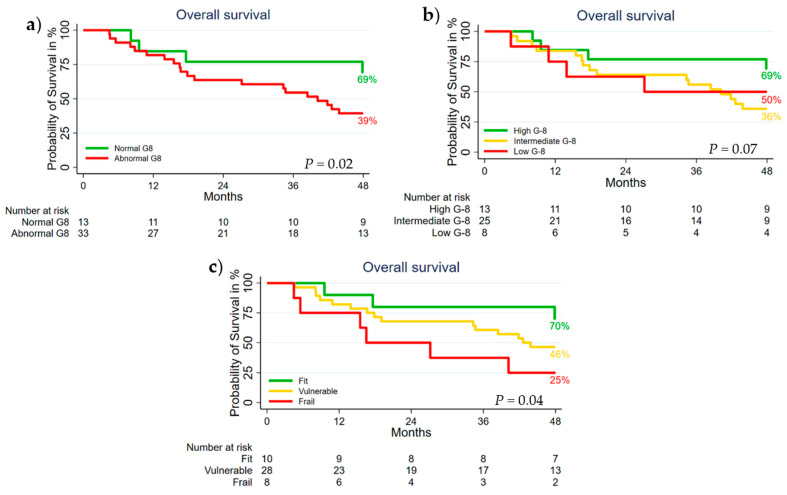
Kaplan–Meier plots of overall survival in (**a**) the normal vs. abnormal G-8 groups, (**b**) the high, intermediate, and low G-8 groups, and (**c**) the fit, vulnerable, and frail G-8 and HGST combination groups.

As illustrated in Figure 1a, the median overall survival (mOS) and the actual 4-year OS for the normal and abnormal G-8 groups was 46 vs. 35 months and 69% vs. 39% (*p* = 0.02), respectively. The mOS of the high, intermediate, and low G-8 groups was 46 vs. 35 vs. 36 months (*p* = 0.07) (Figure 1b). The actual 4-year OS of the high vs. intermediate vs. low G-8 groups was 69% vs. 36% vs. 50%. For the G-8 and HGST combination groups of fit, vulnerable, and frail patients, the mOS was 48 vs. 38 vs. 28 months, and the actual 4-year OS was 70% vs. 46% vs. 25% (*p* = 0.04), respectively (Figure 1c). 

**Figure 2:**
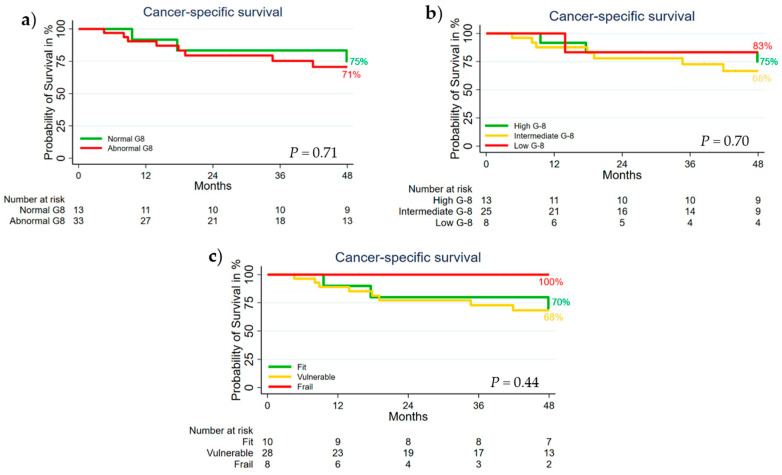
Kaplan–Meier plots of cancer-specific survival in (**a**) the normal vs. abnormal G-8 groups, (**b**) the high, intermediate, and low G-8 groups, and (**c**) the fit, vulnerable, and frail G-8 and HGST combination groups.

No statistically significant differences in CSS were found between the G-8 groups or the G-8 and HGST combination groups (Figure 2). The actual 4-year CSS of the normal vs. abnormal G-8 groups was 75% vs. 71% (*p* = 0.71) (Figure 2a). The actual 4-year CSS of the high vs. intermediate vs. low G-8 groups was 75% vs. 68% vs. 83% (*p* = 0.70) (Figure 2b). The 4-year CSS of the fit vs. vulnerable vs. frail groups was 70%, 68%, and 100% (*p* = 0.44), respectively (Figure 2c). 

Univariate analyses demonstrated that only an age of ≥70 years with a hazard ratio (HR) of 3.10 (95% CI: 1.07-8.99, *p* = 0.04), a G-8 score of ≤14 with an HR of 3.24 (95% CI: 1.12–9.39, *p* = 0.03), and being frail according to the G-8 and HGST combination with an HR of 5.24 (95% CI: 1.34-20.40, *p* = 0.02) were statistical significantly associated with poorer OS. Abnormal HGST scores with an HR of 1.64 (95% CI: 0.72–3.72, *p* = 0.25) demonstrated no statistically significant impacts on OS. In the multivariable model, being vulnerable with a hazard ratio (HR) of 2.03 (95% CI: 0.50–8.12, *p* = 0.32) or frail with an HR of 3.80 (95% CI 0.80–18.01, *p* = 0.09) showed no statistically significant associations with poorer OS compared to being fit (Table 2). The interaction analyses of the Cox multivariable regression model showed no statistically significant interactions between the included variables.

## 4. Discussion

To our knowledge, this is the first study to compare the combination of G-8 and HGST with the G-8 alone in predicting the long-term OS of older patients with NSCLC treated with SBRT. The patients that were identified as frail using the combination of G-8 and HGST had a shorter median OS than the patients with abnormal G-8 scores alone. In a multivariable Cox regression analysis, being identified as vulnerable or frail by the G-8 and HGST combination was associated with poorer long-term OS, though no statistical significance was reached.

In our study, 72% of all patients had a G-8 score of ≤14, consistent with data from previous studies in large populations of older patients with cancer, which reported abnormal G-8 scores in 68–83% of patients [10,13,14,26,27]. However, these studies were conducted in study populations that included various types of cancers, and the majority of the patients were diagnosed with metastatic diseases upon inclusion. In general, patients with advanced cancer may be susceptible to having lower G-8 scores than patients with localized cancer; however, Maebayashi et al. and Cuccia et al. reported abnormal G-8 scores in study populations of older patients with localized NSCLC who were receiving SBRT that were comparable to the scores of patients in our study of 81% and 40%, respectively [28,29]. For both studies, the patients were ≥65 years of age with treatment decisions based on a multidisciplinary assessment and an ECOG-PS of 1 or a Karnofsky performance status of ≥70. No obvious discrepancies in study design or population explained the difference in G-8 scores between the two studies, suggesting that G-8 scores may vary within even a highly specific cancer population. In our study, no lower age limit was chosen for inclusion, and a shift towards more patients with normal G-8 scores might be expected when compared to similar studies due to the risk of including younger patients. Nevertheless, a subanalysis revealed that 73% of patients aged 70 or older in our study had abnormal G-8 scores, showing a similar distribution to that seen across the entire study population. Thus, this study population represented a group of patients that was comparable to the populations of both Maebayashi et al. and Cuccia et al. [28,29]. 

The G-8 is a well-established screening tool in the standard evaluation of older patients with cancer and is known to be a strong predictor of OS [10,30]. In our study, the univariate analysis showed an association between a G-8 score of ≤14 and poorer long-term OS in patients with localized NSCLC treated with SBRT. In a similar study population, Maebayashi et al. reported a statistically significant association between a G-8 score of ≤12 and a poorer long-term OS [28]. Though different G-8 cut-off values were chosen, a significant association between low G-8 scores and poorer long-term OS was found in both studies, thus demonstrating the value of the G-8 screening tool in predicting OS in this population. In previous studies of the G-8 screening tool, a cut-off value of 14 has conventionally been chosen, and this was defined by Bellera et al. as the optimal cut-off value for identifying older patients with cancer who might benefit from a CGA [13,26,30]. However, it has been questioned in recent studies of older patients with cancer whether a G-8 cut-off value at 14 is adequate when evaluating OS [14,15]. Takahashi et al. reported a significant difference in the OS of 264 patients with cancer who were aged ≥70 years when divided into three groups defined by the G-8 cut-off values of <11, 11–14, and >14, suggesting that a new subclassification might lead to more efficient identification of older patients with cancer with poor prognosis [14]. Furthermore, the NORDIC9 trial in 160 patients aged ≥70 years with previously untreated metastatic colorectal cancer showed an association between the G-8 and OS, toxicity, hospitalization, and receiving no more than one cycle of chemotherapy, which became statistically significant when the G-8 cut-off value was lowered to 11 from 14 [15]. Our study found no significant differences between SBRT-treated patients with localized NSCLC when they were divided into three groups using the cut-off values suggested by Takahashi et al. Furthermore, the low G-8 group had longer mOS and 4-year OS than the intermediate G-8 group, suggesting that subdivision of patients with G-8 scores of ≤14 does not give a more precise estimate of OS in patients with localized NSCLC treated with SBRT.

Only sparse information exists on the capability of the HGST as a predictive tool in cancer patients. In non-cancer populations of older people, the HGST is strongly associated with overall muscle strength, physical status, short- and long-term mortality, and morbidity [31,32]. However, the HGST has been reported to be associated with OS in both older patients with newly diagnosed cancer and older patients with advanced cancer [33,34]. Comparably with the findings in our study, Versteeg et al. demonstrated that abnormal HGST was statistically significantly associated with poorer OS (HR of 1.75) in a study population of 103 older patients with advanced cancer [33]. The small sample size in our study is a limitation, and one could speculate that the HGST may be associated with OS in a larger study population of older SBRT-treated NSCLC patients.

A retrospective study by Lycke et al. investigated the added value of an HGST in the prediction of 12-month survival in a population of older patients with cancer and with abnormal G-8 scores [35]. They demonstrated a significant association between abnormal HGST and poorer OS, as well as that the addition of the HGST in G-8 abnormal patients provided a more precise estimate of 12-month OS. In our study, the normal G-8 group and the fit G-8 and HGST combination group had a similar OS; however, when comparing the abnormal G-8 group with the vulnerable and frail G-8 and HGST combination groups, we saw a marked difference in the OS. The abnormal G-8 group had a poorer OS than the vulnerable group but an improved OS compared to the frail group, suggesting, in accordance with the findings of Lycke et al., that the addition of the HGST to the G-8 gives a more precise estimate of OS than that of the G-8 alone. Even though no statistically significant associations were found between OS and the combination of G-8 and HGST in our multivariable Cox regression model, the hazard ratios indicated a strong association with poorer long-term OS. Screenings tools for predicting OS and identifying frailty remain important first steps in addressing age-related comorbidities and optimizing the general health status of older patients with cancer. In patients with localized NSCLC who were receiving SBRT, previous studies have shown that older patients of ≥75 years of age experience the same risk of toxicity and local control rates as those of younger patients [36,37]. Comparably to these studies, only six patients experienced grade 3 toxicity, and no patients experienced grade 4 or 5 toxicity in our study. Definitive SBRT treatment in localized NSCLC offers a more favorable survival benefit of approximately three years in favor of SBRT in comparison with similar patients without active treatment, and a recent study by Klement et al. suggested that all patients with localized NSCLC should be offered SBRT treatment, irrespectively of their comorbidity status [8,38,39,40]. In agreement with a previous study in SBRT-treated NSCLC patients by Franco et al., our CSS findings suggest, though not statistically significant, that the majority of these patients died of other causes than lung cancer, and the frail G-8 and HGST group was at the highest risk [41]. In our study, none of the frail G-8 and HGST combination group patients died of lung cancer, demonstrating the importance of a future focus on optimizing age-related comorbidities in older SBRT-treated patients with localized NSCLC. In this study, univariate and multivariate analyses indicated the improved OS in favor of patients receiving CGA. However, no statistically significant impacts on OS were found in either model, which could be due to the small sample size of the study and can only be confirmed in a larger patient population.

We suggest that the combination of the G-8 and HGST is a valuable tool when evaluating older SBRT-treated patients with localized NSCLC in order to help guide clinicians in optimizing individual patients’ general health status.

## 5. Conclusions

This study suggests that there might be a benefit of adding a physical test to the G-8 for more precisely predicting overall survival in SBRT-treated patients with localized NSCLC. However, this should be confirmed in a larger study population.

## Figures and Tables

**Table 1:** Baseline characteristics of patients with localized non-small cell lung cancer who were treated with stereotactic body radiation therapy.

Patient Characteristics	Patients (*n* = 46)
**Sex**	
Female	25 (54%)
Male	21 (46%)
**Age (years)**	
Median (range)	72 (52–87)
≥70 years	33 (72%)
**Reason of SBRT referral**	
Unfit for surgery ^1^	42 (91%)
Declined surgery	4 (9%)
**Histology**	
Adenocarcinoma	23 (50%)
Non-adenocarcinoma	23 (50%)
**Lung cancer stage**	
1A	30 (65%)
1B	15 (33%)
Synchronous NSCLC	1 (2%)
**Prescribed radiation dose**	
45Gy/3F (BED 112Gy)	4 (9%)
66Gy/3F (BED 211Gy)	42 (91%)
**ECOG Performance Status**	
0–1	28 (61%)
≥2	18 (39%)
**Charlson Comorbidity Index**	
0–1	19 (41%)
2–3	21 (46%)
>3	6 (13%)
**Barthel-20**	
Normal (20–19)	32 (70%)
Disability (≤18)	14 (30%)
**G-8 total (Abnormal ≤14)**	
High (>14)	13 (28%)
Intermediate (11–14)	25 (54%)
Low (<11)	8 (18%)
**CST (*n* = 13)**	
Normal (≥10)	3 (23%)
Abnormal (<10)	10 (77%)
**HGST ^2^**	
Normal (♂ ≥ 21 kg/♀ ≥ 15 kg)	35 (76%)
Abnormal (♂ < 21 kg/♀ < 15 kg)	11 (24%)
**G-8 + HGST**	
Fit (normal G-8 and HGST)	10 (22%)
Vulnerable (abnormal G-8 or abnormal HGST)	26 (61%)
Frail (abnormal G-8 and HGST)	8 (17%)
**CGA ^3^**	
Yes	24 (50%)
No	22 (48%)

ECOG Performance Status—Eastern Cooperative Oncology Group’s definition of performance status, G-8—Geriatric 8 screening tool, CST—Chair-stand test, HGST—Hand-grip strength test, ♂—male, ♀—female, G-8 + HGST—Combination of Geriatric 8 and hand-grip strength test, CGA—Comprehensive Geriatric Assessment. ^1^ Unfit for surgery encompasses patients considered medically inoperable or best served with SBRT. ^2^ Hand-grip strength measured using a Smedley Dynamometer. ^3^ Patients were randomized 1:1 to receive CGA or not upon inclusion in the previous randomized study.

**Table 2:** Univariate analysis and Cox multivariate regression model in patients with localized non-small cell lung cancer who were treated with stereotactic body radiation therapy.

Patient Characteristics	Univariate Model			Multivariate Model		
	HR	*p*-Value	95% CI	HR	*p*-Value	95%CI
Male gender	1.55	0.26	0.72–3.32	1.35	0.49	0.58–3.15
Age ≥70 years	3.10	0.04	1.07–8.99	3.28	0.07	0.92–11.78
Adenocarcinoma	0.65	0.27	0.31–1.39	1.00	0.99	0.45–2.26
G-8 score ≤14	3.24	0.03	1.12–9.39			
HGST score ♂ < 21 kg/♀ < 15 kg ^1^	1.64	0.24	0.72–3.72			
HGST+G-8 combination ^2^						
Fit (reference)	-	-	-	-	-	-
Vulnerable	2.77	0.10	0.81–9.44	2.03	0.32	0.50–8.18
Frail	5.24	0.02	1.34–20.40	3.80	0.09	0.80–18.01
ECOG Performance Status ≥2	1.94	0.08	0.92–4.08	1.12	0.84	0.37–3.40
Charlson Comorbidity Index						
0–1 (reference)	-	-	-	-	-	-
2–3	1.89	0.13	0.83–4.33	1.45	0.42	0.59–3.56
>3	1.92	0.28	0.58–6.32	3.89	0.06	0.96–15.81
Barthel-20 score <19	1.56	0.26	0.72–3.40	1.06	0.92	0.35–3.17
CGA ^3^	0.66	0.28	0.31–1.40	0.70	0.44	0.28–1.75

G-8—Geriatric 8 screening tool, HGST—hand-grip strength test, ♂—male, ♀—female, ECOG Performance Status—Eastern Cooperative Oncology Group’s definition of performance status. ^1^ Hand-grip strength test measured using a Smedley Dynamometer. ^2^ Fit (normal G-8 and normal HGST), Vulnerable (abnormal G-8 or abnormal HGST), and Frail (abnormal G-8 and abnormal HGST). ^3^ Patients were randomized 1:1 to receive CGA or not upon inclusion in the previous randomized study.

## Data Availability

The data presented in this study are available in this article.

## Appendix A

**Table A1:** Baseline characteristics of patients with localized non-small cell lung cancer who were treated with stereotactic body radiation therapy and divided into three groups: normal vs. abnormal G-8 scores, high vs. intermediate vs. low G-8 scores, and fit vs. vulnerable vs. frail G-8+HGST scores.

Patient Characteristics	G-8, 2 Groups		G-8, 3 Groups ^1^			G-8 + HGST ^2^		
	Normal	Abnormal	High	Intermediate	Low	Fit	Vulnerable	Frail
**Sex**								
Female	4 (31%)	21 (64%)	4 (31%)	17 (68%)	4 (50%)	6 (60%)	10 (36%)	5 (62.5%)
Male	9 (69%)	12 (36%)	9 (69%)	8 (32%)	4 (50%)	4 (40%)	18 (64%)	3 (37.5%)
**Age**								
Median (range)	72 (52–79)	72 (56–87)	72 (52–79)	72 (56–87)	71 (57–83)	72 (52–79)	73 (58–87)	71 (57–80)
≥70 years (%)	9 (69%)	25 (74%)	9 (69%)	19 (76%)	5 (63%)	6 (60%)	21 (75%)	6 (75%)
**Reason of SBRT referral**								
Unfit for surgery ^3^	13 (100%)	29 (88%)	13 (100%)	22 (88%)	7 (88%)	10 (100%)	26 (93%)	6 (75%)
Declined surgery	0 (0%)	4 (12%)	0 (0%)	3 (12%)	1 (12%)	0 (0%)	2 (7%)	2 (25%)
**Lung cancer stage**								
1A	8 (61%)	22 (68%)	8 (61%)	16 (64%)	6 (75%)	6 (60%)	18 (64%)	6 (75%)
1B	4 (31%)	11 (32%)	4 (31%)	9 (36%)	2 (25%)	3 (30%)	10 (36%)	2 (25%)
Synchronous NSCLC	1 (8%)	0 (0%)	1 (8%)	0 (0%)	0 (0%)	1 (10%)	0 (0%)	0 (0%)
**Histology**								
Adeno	7 (54%)	16 (47%)	7 (54%)	11 (44%)	5 (63%)	7 (70%)	12 (43%)	4 (50%)
Non-adeno	6 (46%)	18 (53%)	6 (46%)	14 (56%)	3 (37%)	3 (30%)	16 (57%)	4 (50%)
**Prescribed radiation dose**								
45Gy/3F(BED 112Gy)	2 (15%)	2 (6%)	2 (15%)	2 (8%)	0 (0%)	2 (20%)	2 (7%)	0 (0%)
66Gy/3F (BED 211Gy)	11 (85%)	31 (94%)	11 (85%)	23 (92%)	8 (100%)	8 (80%)	26 (93%)	8 (100%)
**ECOG Performance status**								
0–1	11 (85%)	17 (50%)	11 (85%)	14 (56%)	3 (37%)	9 (90%)	16 (57%)	3 (37%)
≥2	2 (15%)	17 (50%)	2 (15%)	11 (44%)	5 (63%)	1 (10%)	12 (43%)	5 (65%
**CCI**								
0–1	7 (54%)	13 (38%)	7 (54%)	9 (36%)	3 (37%)	6 (60%)	10 (36%)	3 (37%)
2–3	4 (31%)	17 (50%)	4 (31%)	13 (52%)	4 (50%)	2 (20%)	14 (50%)	5 (63%)
>3	2 (15%)	4 (12%)	2 (15%)	3 (12%)	1 (13%)	2 (20%)	4 (14%)	0 (0%)
**Barthel 20**								
Normal (20–19)	10 (77%)	22 (65%)	10 (77%)	19 (79%)	3 (37%)	8 (80%)	20 (71%)	4 (50%)
Disability (≤18)	3 (23%)	12 (35%)	3 (23%)	6 (24%)	5 (63%)	2 (20%)	8 (29%)	4 (50%)
**G-8 total**								
Normal (>14)	13 (100%)	0 (0%)	13 (100%)	0 (0%)	0 (0%)	10 (100%)	3 (11%)	0 (0%)
Abnormal (≤14)	0 (0%)	43 (100%)	0 (0%)	25 (100%)	8 (100%)	0 (0%)	25 (89%)	8 (100%)
**CST**								
Normal (≥10)	1 (25%)	2 (22%)	1 (25%)	1 (12%)	1 (100%)	0 (0%)	3 (27%)	0 (0%)
Abnormal (<10)	3 (75%)	7 (78%)	3 (75%)	7 (88%)	0 (0%)	2 (100%)	8 (73%)	0 (0%)
**HGST**								
Normal (♂ ≥ 21 kg/♀ ≥ 15 kg)	10 (77%)	25 (76%)	10 (77%)	20 (80%)	5 (63%)	10 (100%)	25 (89%)	0 (0%)
Abnormal (♂ < 21 kg/♀ < 15 kg)	3 (23%)	8 (24%)	3 (23%)	5 (20%)	3 (37%)	0 (0%)	3 (11%)	8 (100%)
**CGA ^4^**								
Yes	6 (46%)	19 (56%)	6 (46%)	15 (60%)	3 (37%)	6 (60%)	12 (43%)	4 (50%)
No	7 (54%)	15 (44%)	7 (54%)	10 (40%)	5 (63%)	4 (40%)	16 (57%)	4 (50%)

ECOG Performance Status—Eastern Cooperative Oncology Group’s definition of performance status, G-8—Geriatric 8 screening tool, CST—Chair-stand test, HGST—Hand-grip strength test, ♂—male, ♀—female, G-8 + HGST—Combination of Geriatric 8 and hand-grip strength test, CGA—Comprehensive Geriatric Assessment. ^1^ High G-8 score (>14), Intermediate G-8 score (11–14), and Low G-8 score (<11). ^2^ Geriatric 8 screening tool and hand-grip strength test combination. Hand-grip strength was measured using a Smedley Dynamometer. Fit (normal G-8 and normal HGST), Vulnerable (abnormal G-8 or abnormal HGST), and Frail (abnormal G-8 and abnormal HGST). ^3^ Unfit for surgery encompasses patients considered medically inoperable or best served with SBRT. ^4^ Patients were randomized 1:1 to receive CGA or not upon inclusion in the previous randomized study. A statistically significant difference was found in ECOG-PS scores between patients with normal vs. abnormal G-8 scores (*p* = 0.05). Otherwise, no statistically significant differences were observed between the two G-8 groups, the three G-8 groups, or the fit, vulnerable, or frail combination groups.

## References

[1] Sung H., Ferlay J., Siegel R.L., Laversanne M., Soerjomataram I., Jemal A., Bray F. (2021). Global cancer statistics 2020: GLOBOCAN estimates of incidence and mortality worldwide for 36 cancers in 185 countries. CA Cancer J. Clin..

[2] Divo M.J., Martinez C.H., Mannino D.M. (2014). Ageing and the epidemiology of multimorbidity. Eur. Respir. J..

[3] Owonikoko T.K., Ragin C.C., Belani C.P., Oton A.B., Gooding W.E., Taioli E., Ramalingam S.S. (2007). Lung cancer in elderly patients: An analysis of the surveillance, epidemiology, and end results database. J. Clin. Oncol..

[4] Donington J., Ferguson M., Mazzone P., Handy J., Schuchert M., Fernando H., Loo B., Lanuti M., de Hoyos A., Detterbeck F. (2012). American College of Chest Physicians and Society of Thoracic Surgeons consensus statement for evaluation and management for high-risk patients with stage I non-small cell lung cancer. Chest.

[5] Nyman J., Hallqvist A., Lund J.A., Brustugun O.T., Bergman B., Bergstrom P., Friesland S., Lewensohn R., Holmberg E., Lax I. (2016). SPACE—A randomized study of SBRT vs conventional fractionated radiotherapy in medically inoperable stage I NSCLC. Radiother. Oncol..

[6] Guckenberger M., Andratschke N., Dieckmann K., Hoogeman M.S., Hoyer M., Hurkmans C., Tanadini-Lang S., Lartigau E., Mendez Romero A., Senan S. (2017). ESTRO ACROP consensus guideline on implementation and practice of stereotactic body radiotherapy for peripherally located early stage non-small cell lung cancer. Radiother. Oncol..

[7] Soto-Perez-de-Celis E., Li D., Yuan Y., Lau Y.M., Hurria A. (2018). Functional versus chronological age: Geriatric assessments to guide decision making in older patients with cancer. Lancet Oncol..

[8] Jeppesen S.S., Hansen N.C.G., Schytte T., Hansen O. (2018). Survival of localized NSCLC patients without active treatment or treated with SBRT. Acta Oncol..

[9] Martinez-Tapia C., Paillaud E., Liuu E., Tournigand C., Ibrahim R., Fossey-Diaz V., Culine S., Canoui-Poitrine F., Audureau E., Group E.S. (2017). Prognostic value of the G8 and modified-G8 screening tools for multidimensional health problems in older patients with cancer. Eur. J. Cancer.

[10] Soubeyran P., Bellera C., Goyard J., Heitz D., Cure H., Rousselot H., Albrand G., Servent V., Jean O.S., van Praagh I. (2014). Screening for vulnerability in older cancer patients: The ONCODAGE Prospective Multicenter Cohort Study. PLoS ONE.

[11] Pottel L., Lycke M., Boterberg T., Pottel H., Goethals L., Duprez F., Rottey S., Lievens Y., Van Den Noortgate N., Geldhof K. (2015). G-8 indicates overall and quality-adjusted survival in older head and neck cancer patients treated with curative radiochemotherapy. BMC Cancer.

[12] Extermann M., Aapro M., Bernabei R., Cohen H.J., Droz J.P., Lichtman S., Mor V., Monfardini S., Repetto L., Sorbye L. (2005). Use of comprehensive geriatric assessment in older cancer patients: Recommendations from the task force on CGA of the International Society of Geriatric Oncology (SIOG). Crit. Rev. Oncol. Hematol..

[13] Bellera C.A., Rainfray M., Mathoulin-Pelissier S., Mertens C., Delva F., Fonck M., Soubeyran P.L. (2012). Screening older cancer patients: First evaluation of the G-8 geriatric screening tool. Ann. Oncol..

[14] Takahashi M., Takahashi M., Komine K., Yamada H., Kasahara Y., Chikamatsu S., Okita A., Ito S., Ouchi K., Okada Y. (2017). The G8 screening tool enhances prognostic value to ECOG performance status in elderly cancer patients: A retrospective, single institutional study. PLoS ONE.

[15] Winther S.B., Liposits G., Skuladottir H., Hofsli E., Shah C.H., Poulsen L.O., Ryg J., Osterlund P., Berglund A., Qvortrup C. (2019). Reduced-dose combination chemotherapy (S-1 plus oxaliplatin) versus full-dose monotherapy (S-1) in older vulnerable patients with metastatic colorectal cancer (NORDIC9): A randomised, open-label phase 2 trial. Lancet Gastroenterol. Hepatol..

[16] Rassam Y., Schindler A., Willschrei P., Horstmann M. (2020). The G8 questionnaire as a geriatric screening tool in urooncology. Aktuelle Urol..

[17] Bruijnen C.P., van Harten-Krouwel D.G., Koldenhof J.J., Emmelot-Vonk M.H., Witteveen P.O. (2019). Predictive value of each geriatric assessment domain for older patients with cancer: A systematic review. J. Geriatr. Oncol..

[18] Bohannon R.W. (2015). Muscle strength: Clinical and prognostic value of hand-grip dynamometry. Curr. Opin. Clin. Nutr. Metab. Care.

[19] Jeppesen S.S., Matzen L.E., Brink C., Bliucukiene R., Kasch S., Schytte T., Kristiansen C., Hansen O. (2018). Impact of comprehensive geriatric assessment on quality of life, overall survival, and unplanned admission in patients with non-small cell lung cancer treated with stereotactic body radiotherapy. J. Geriatr. Oncol..

[20] Roberts H.C., Denison H.J., Martin H.J., Patel H.P., Syddall H., Cooper C., Sayer A.A. (2011). A review of the measurement of grip strength in clinical and epidemiological studies: Towards a standardised approach. Age Ageing.

[21] Frederiksen H., Hjelmborg J., Mortensen J., McGue M., Vaupel J.W., Christensen K. (2006). Age trajectories of grip strength: Cross-sectional and longitudinal data among 8,342 Danes aged 46 to 102. Ann. Epidemiol..

[22] Csuka M., McCarty D.J. (1985). Simple method for measurement of lower extremity muscle strength. Am. J. Med..

[23] Oken M.M., Creech R.H., Tormey D.C., Horton J., Davis T.E., McFadden E.T., Carbone P.P. (1982). Toxicity and response criteria of the Eastern Cooperative Oncology Group. Am. J. Clin. Oncol..

[24] Charlson M.E., Pompei P., Ales K.L., MacKenzie C.R. (1987). A new method of classifying prognostic comorbidity in longitudinal studies: Development and validation. J. Chronic Dis..

[25] Collin C., Wade D.T., Davies S., Horne V. (1988). The Barthel ADL Index: A reliability study. Int. Disabil. Stud..

[26] Kenis C., Decoster L., Van Puyvelde K., De Greve J., Conings G., Milisen K., Flamaing J., Lobelle J.P., Wildiers H. (2014). Performance of two geriatric screening tools in older patients with cancer. J. Clin. Oncol..

[27] Liuu E., Canoui-Poitrine F., Tournigand C., Laurent M., Caillet P., Le Thuaut A., Vincent H., Culine S., Audureau E., Bastuji-Garin S. (2014). Accuracy of the G-8 geriatric-oncology screening tool for identifying vulnerable elderly patients with cancer according to tumour site: The ELCAPA-02 study. J. Geriatr. Oncol..

[28] Maebayashi T., Ishibashi N., Aizawa T., Sakaguchi M., Saito T., Kawamori J., Tanaka Y. (2018). Significance of stereotactic body radiotherapy in older patients with early stage non-small cell lung cancer. J. Geriatr. Oncol..

[29] Cuccia F., Mortellaro G., Mazzola R., Donofrio A., Valenti V., Tripoli A., Matranga D., Lo Casto A., Failla G., Di Miceli G. (2020). Prognostic value of two geriatric screening tools in a cohort of older patients with early stage Non-Small Cell Lung Cancer treated with hypofractionated stereotactic radiotherapy. J. Geriatr. Oncol..

[30] Decoster L., Van Puyvelde K., Mohile S., Wedding U., Basso U., Colloca G., Rostoft S., Overcash J., Wildiers H., Steer C. (2015). Screening tools for multidimensional health problems warranting a geriatric assessment in older cancer patients: An update on SIOG recommendations. Ann. Oncol..

[31] Rijk J.M., Roos P.R., Deckx L., van den Akker M., Buntinx F. (2016). Prognostic value of handgrip strength in people aged 60 years and older: A systematic review and meta-analysis. Geriatr. Gerontol. Int..

[32] Norman K., Stobaus N., Gonzalez M.C., Schulzke J.D., Pirlich M. (2011). Hand grip strength: Outcome predictor and marker of nutritional status. Clin. Nutr..

[33] Versteeg K.S., Blauwhoff-Buskermolen S., Buffart L.M., de van der Schueren M.A.E., Langius J.A.E., Verheul H.M.W., Maier A.B., Konings I.R. (2018). Higher Muscle Strength Is Associated with Prolonged Survival in Older Patients with Advanced Cancer. Oncologist.

[34] Kilgour R.D., Vigano A., Trutschnigg B., Lucar E., Borod M., Morais J.A. (2013). Handgrip strength predicts survival and is associated with markers of clinical and functional outcomes in advanced cancer patients. Support. Care Cancer.

[35] Lycke M., Ketelaars L., Martens E., Lefebvre T., Pottel H., Van Eygen K., Cool L., Pottel L., Kenis C., Schofield P. (2019). The added value of an assessment of the patient’s hand grip strength to the comprehensive geriatric assessment in G8-abnormal older patients with cancer in routine practice. J. Geriatr. Oncol..

[36] Mancini B.R., Park H.S., Harder E.M., Rutter C.E., Corso C.D., Decker R.H., Husain Z.A. (2016). Elderly patients undergoing SBRT for inoperable early-stage NSCLC achieve similar outcomes to younger patients. Lung Cancer.

[37] Kreinbrink P., Blumenfeld P., Tolekidis G., Sen N., Sher D., Marwaha G. (2017). Lung stereotactic body radiation therapy (SBRT) for early-stage non-small cell lung cancer in the very elderly (>/=80years old): Extremely safe and effective. J. Geriatr. Oncol..

[38] Detterbeck F.C., Gibson C.J. (2008). Turning gray: The natural history of lung cancer over time. J. Thorac. Oncol..

[39] Wao H., Mhaskar R., Kumar A., Miladinovic B., Djulbegovic B. (2013). Survival of patients with non-small cell lung cancer without treatment: A systematic review and meta-analysis. Syst. Rev..

[40] Klement R.J., Belderbos J., Grills I., Werner-Wasik M., Hope A., Giuliani M., Ye H., Sonke J.J., Peulen H., Guckenberger M. (2016). Prediction of Early Death in Patients with Early-Stage NSCLC-Can We Select Patients without a Potential Benefit of SBRT as a Curative Treatment Approach?. J. Thorac. Oncol..

[41] Franco I., Chen Y.H., Chipidza F., Agrawal V., Romano J., Baldini E., Chen A., Colson Y., Hou Y., Kozono D. (2018). Use of frailty to predict survival in elderly patients with early stage non-small-cell lung cancer treated with stereotactic body radiation therapy. J. Geriatr. Oncol..

